# Efficient and Safe Editing of Porcine Endogenous Retrovirus Genomes by Multiple-Site Base-Editing Editor

**DOI:** 10.3390/cells11243975

**Published:** 2022-12-08

**Authors:** Shuwen Zheng, Haiwen Zhong, Xiaoqing Zhou, Min Chen, Wansheng Li, Yin Zi, Yue Chi, Jinling Wang, Wei Zheng, Qingjian Zou, Liangxue Lai, Chengcheng Tang

**Affiliations:** 1 Guangdong Provincial Key Laboratory of Large Animal Models for Biomedicine, School of Biotechnology and Health Science, Wuyi University, Jiangmen 529020, China; 2 College of Animal Science, South China Agricultural University, Guangzhou 510642, China; 3 Key Laboratory of Regenerative Biology, South China Institute for Stem Cell Biology and Regenerative Medicine, Guangzhou Institutes of Biomedicine and Health, Chinese Academy of Sciences, Guangzhou 510530, China

**Keywords:** porcine endogenous retroviruses, base editors, multiple loci

## Abstract

Gene-modified miniature pigs serve as alternative tissue and organ donors for xenotransplantation to alleviate the shortage of human allogenic organs. However, the high copy number of porcine endogenous retrovirus (PERV) genomes integrates with the porcine genome, which has a potential risk of cross-species transmission and hinders the clinical practice of xenotransplantation. Recently, CRISPR/Cas9 has been used to inactivate PERVs. However, Cas9 also triggers severe DNA damage at multiple integrated PERV sites in the porcine genome, which induces senescence and apoptosis of porcine cells. In this study, the cytosine base editor (CBE), an efficient and safe editor that does not cause DNA double strand breaks (DSBs), was used for PERV editing to reduce cytotoxic effects. Seven sgRNAs were set to target *gag* and *pol* loci of PERVs to induce premature stop codons. We found that approximately 10% of cell clones were completely inactivated for PERVs in pig ST cells, and the plasmid that was used for editing the PERVs did not integrate into host genome and influence the karyotype of the modified cells. Our studies offer a powerful and safe strategy for further generating PERV-knockout pigs using base editors.

## 1. Introduction

Xenotransplantation using cells, tissues or organs from pigs into humans has been considered as a potential solution to organ shortages [1]. However, the clinical use of porcine organs has been hindered by immunological rejection, histoincompatibilities [2] and the concern of transmission of pig viruses to humans [3,4]. Recently, humanized PERVKO·3 KO·9 TG pigs were generated. Their cells were resistant to human humoral rejection, cell-mediated damage and pathogenesis associated with dysregulated coagulation [5]. The heart of an engineered pig has been transplanted into a dying patient and it pumped in the new host, indicating that the transplantation of organs from pig to human can be realized. However, the patient died two months later after his transplanted surgery; porcine cytomegalovirus (PCMV) infection may have contributed to his death [6]. Although there are no preclinical or clinical trials demonstrating that PERVs can infect humans, it was reported that PERVs were able to infect human cell lines and primary cells [7,8], increasing the potential PERV infection risk in pig-to-human transplantation.

Strategies such as a PERV-specific vaccine [9,10,11], antiretroviral drugs [12,13], PERV-specific siRNAs [14,15] and gene editors [16,17,18] have been used to reduce the risk of PERV transmission. Gene editors are the tools with the greatest potential for completely solving PERV transmission. However, dozens of porcine endogenous retroviruses (PERVs) are distributed throughout the pig genome [19]. PERV genome targeting using ZFN or CRISPR/Cas9 would produce numerous double-stranded breaks (DSBs) in the pig genome, causing cell senescence, apoptosis and other cytotoxic effects [16,17,18].

Recently, the cytosine base editor (CBE) has been used to convert C to T with high efficiency without causing DSBs [20]. This technique has been developed to silence endogenous genes through directly induced nonsense mutations [21,22], which is much safer than ZFN or Cas9. In this study, to disrupt PERVs efficiently and thoroughly, we used the base editor BE4 max to disrupt PERVs by introducing a premature stop codon at seven sites in the *gag* and *pol* region of PERVs.

## 2. Materials and Methods

### 2.1. Plasmids and Oligonucleotides

The multiplex sgRNAs were fused by overlapping PCR to obtain 3 sgRNA tandem structures, gtRNA–gag, gtRNA–pol1 and gtRNA–pol2. The primers are listed in Supplementary Table S1. The PCR products were then cloned into the BbsI-digested U6–sgRNA cloning vector to obtain the sgRNA-expressing plasmids pU6–gtRNA–gag, pU6–gtRNA–pol1, and pU6–gtRNA–pol2. The sequence of the seven tandem sgRNAs: U6–gtRNA–gag, U6–gtRNA–pol1 and U6–gtRNA–pol2 are listed in Supplementary Table S2. Then, the 7 sgRNA expression elements were introduced into the MluI-cleaved pCMV-BE4 max-P2 A-Puro vector using recombination kit (ClonExpress^®^ MultiS, Vazyme, Gaithersburg, MD, USA) to obtain the pCMV-BE4 max-P2 A-Puro-tandem sgRNA plasmid. Finally, all expressed elements such as the 7 sgRNAs, BE4 max and the puromycin resistance gene were cloned into the middle of pCEP4 by HindIII and PmeI to obtain the pCEP4-BE4 max-P2 A-Puro-tandem sgRNAs, which were also named MAIO-epiCBE. The procedure for the construction of the MAIO-epiCBE plasmid is shown in Supplementary Figure S1.

### 2.2. Cell Culture and Transfection

The ST cell lines were fibroblast-like cells that were isolated from the testis of a male pig and were purchased from Procell (CL0219). This cell line was cultured in Dulbecco’s modified Eagle’s medium (DMEM, HyClone, Waltham, MA, USA) supplemented with 10% fetal bovine serum (FBS, Gibco), 1% nonessential amino acids (NEAA, Gibco, Waltham, MA, USA), 2 mM GlutaMAX (Gibco), and 1 mM sodium pyruvate (Gibco). All cells were cultured at 37 °C with 5% CO_2_. ST cells were cultured in 10 cm dishes and transfected the next day at approximately 70% confluency. For electroporation, the cells were digested with 0.05% trypsin (Gibco) and collected by centrifugation. Then, the collected cells were electroporated with MAIO-epiCBE (30 μg) at 1230 V, 10 ms, and 3 pulses using the Neon™ transfection system (Life Technology, Carlsbad, CA, USA). Cells transfected with null (the null sgRNA sequence was as follow: CGCTTCCGCGGCCCGTTCAA) were used as a negative control.

### 2.3. Cell Lysis and Genomic PCR

The cells were sub-cultured at a split ratio of 1:20 after one day of electroporation. After selecting with puromycin for about 15 days, single cell colonies were picked and cultured on 24-well plates. When the cells reached approximately 90% confluency, cell colonies were sub-cultured on 12-well plates, and 20% of an individual cell colony was collected and lysed in 10 μL of the lysis buffer (0.45% NP-40 + 0.6% proteinase K) for 60 min at 56 °C, followed by 10 min at 95 °C. Then the lysates were used as a template for PCR. The primers used for amplification of the *gag* region were gag-F1 and gag-R1. The primers used for amplification of the *pol* region were pol-F1 and pol-R1. Sequencing of PCR products was performed to determine mutation patterns. Colonies of cells with the expected mutations were then frozen in cell-freezing medium (90% fetal bovine serum and 10% dimethyl sulfoxide) for further analysis.

### 2.4. Sanger Sequencing

DNA was extracted from the transfected cells and the target regions were amplified using the appropriate site-specific primers. Each PCR was performed in 30 μL volume comprising 1 μL of the template, 1 μL of each 10 μM primer, and 15 μL 2× Rapid Taq Master Mix (Vazyme) with the following thermal cycler conditions: 95 °C for 3 min; 30 cycles of 95 °C for 15 s, 55 °C for 15 s, and 72 °C for 10 s; followed by 72 °C for 5 min as a final extension; and held at 12 °C. The PCR products were sequenced to identify point mutations by IGE Biotech (Guangzhou). Primers used for PCR were also used for sequencing and are listed in Supplementary Table S3. The base-editing efficiency was quantified using *EditR* (http://baseeditr.com).

### 2.5. Mi-seq Sequencing with Hi-TOM Platform

For the Mi-seq sequencing, PCR was performed in 50 μL volumes comprising 1 μL of the template, 1.5 μL of each 10 μM primer, and 25 μL 2× KOD OneTM PCR master mix with the following thermal cycler conditions: 98 °C for 3 min; 30 cycles of 98 °C for 10 s, 60 °C for 5 s, and 68 °C for 1 s; followed by 72 °C for 5 min as a final extension; and held at 12 °C. The primers used for amplification of the *gag* region were M-gag-F1 and M-gag-R1. The primers used for amplification of the *pol* region were M-pol-F1 and M-pol-R1. All the amplified products in different target regions were separated in 1.5% agarose gels with 1× TAE buffer, and the target bands were cut for extraction using a HiPure Gel Pure DNA Mini kit (Magen). The purified DNA products were deep-sequenced and assayed using Hi-TOM platform [23]. The protospacer sequences in the reads were investigated to identify C-to-T and G-to-A point mutations, and indels. Primers used for genomic PCR and sequencing are listed in Supplementary Table S3.

### 2.6. Calculating the Results of Hi-TOM Platform

For each ST cell clone, the Hi-TOM platform sequencing results can be divided into 4 types (Supplementary Figure S2). The base-editing efficiency of *gag* region in PERV is equal to 100% minus the editing efficiency of the “no expected editing” type (Supplementary Figure S2). The algorithm of the editing efficiency of *pol* region is the same as that of the *gag* region.

### 2.7. Detection of Integration Status of the MAIO-epiCBE System

The transfected monoclonal cell lines were diluted to a low cell density and cultured with puromycin-free medium for 15 days to form single cell clones. The positive clones with PERV modification were selected for genome extraction and PCR to identify the presence of the base-editor system. The primer pair episomal-F and episomal-R was used to amplify the ampicillin gene in the MAIO-epiCBE plasmid. The expected product size was 239 bp. The sequence of episomal-F and episomal-R is listed in Supplementary Table S3.

### 2.8. Co-Culture of ST Cells to HEK293-GFP and Droplet Digital PCR

The HEK293-GFP and ST cells were co-cultured in a 100 mm dish with 1:1 ratio for 7 days. Then the HEK293-GFPs were isolated from the co-cultures by FACS sorting. The genomic DNA of these sorted cells were used for droplet digital PCR (ddPCR). First, 100 ng genomic DNA were digested with MseI (10 U) at 37 °C for 2 h, then heat inactivated at 65 °C for 20 min. DdPCR was performed by RainSure Scientific company (Suzhou, China). Cycling conditions were used as follows: the enzyme was activated at 95 °C for 12 min; 40 cycles at 94 °C for 45 s and 59 °C for 90 s; the enzyme was deactivated at 98 °C for 10 min. The primers used for ddPCR are listed in Supplementary Table S4.

### 2.9. Karyotyping Analysis

The karyotyping analysis was performed by Hangzhou Kayotapu Biological Technology Co., Ltd. (Hangzhou, China).

## 3. Results

### 3.1. Establishment of an OriP/EBNA1-Based All-in-One Plasmid Expressing CBE and Seven sgRNAs

**Figure 1:**
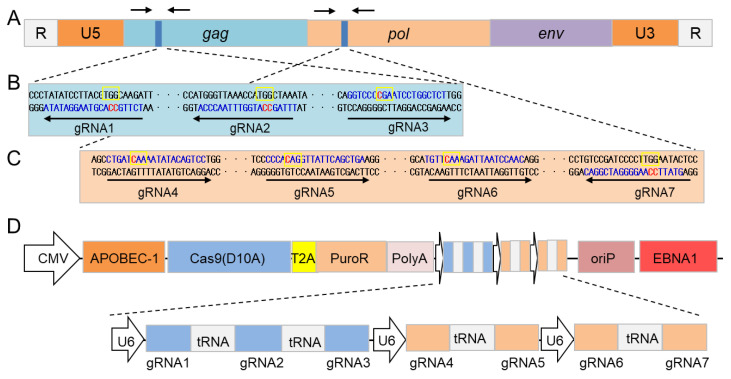
The design of MAIO-epiCBE system for base editing of the PERV genome. (**A**) Schematic diagram of PERV structure. (**B**) Three sgRNAs targeting the *gag* region. (**C**) Four sgRNAs targeting the *pol* region. The sgRNA target sequences are in blue. The objective C base is marked in red. The target codons are boxed in a yellow frame. (**D**) Structure of multi-target all-in-one Or-iP/EBNA1-based CBE vector.

Despite the highly conserved genome of PERVs, virus variation occurs during long periods of symbiotic co-existence of a virus and its host. To target the entire genome of a PERV, seven sgRNAs were designed to target the regions of *gag* and *pol*, respectively (Figure 1A). Among them, three were designed to target the *gag* region on codons TGG, TGG and CGA (Figure 1B) and the other four were designed to target the *pol* region on codons CAA, CAG, CAA and TGG (Figure 1C).

Previous work had shown that the transfection of an all-in-one vector [24] or the persistent expression of Cas9 and sgRNA by an OriP/EBNA1-based episome [25,26] can increase the gene-editing efficiency. Therefore, we designed a multi-target all-in-one OriP/EBNA1-based CBE vector, named MAIO-epiCBE (Figure 1D). The MAIO-epiCBE consists of OriP/EBNA1-based episomes, seven sgRNAs targeting different regions of pig PERVs, CBE and the puromycin resistance gene. The OriP/EBNA1-based episomes can drive plasmid duplication once per cell division, allowing CBE and sgRNAs to be persistently expressed in cells [27]; seven sgRNAs targeting different regions of pig PERVs can reduce the possible influence of PERV mutations and increase the editing efficiency of PERVs; the puromycin resistance gene allows the selection of transfected cells and the maintenance of the expression of the component of CBE and sgRNAs. After withdrawal of the puromycin, the episomal-based MAIO-epiCBE vector becomes lost after several cell divisions [28], allowing the removal of exogenous genes.

### 3.2. Gag and Pol of PERV Edited by MAIO-epiCBE with High Efficiency

**Figure 2:**
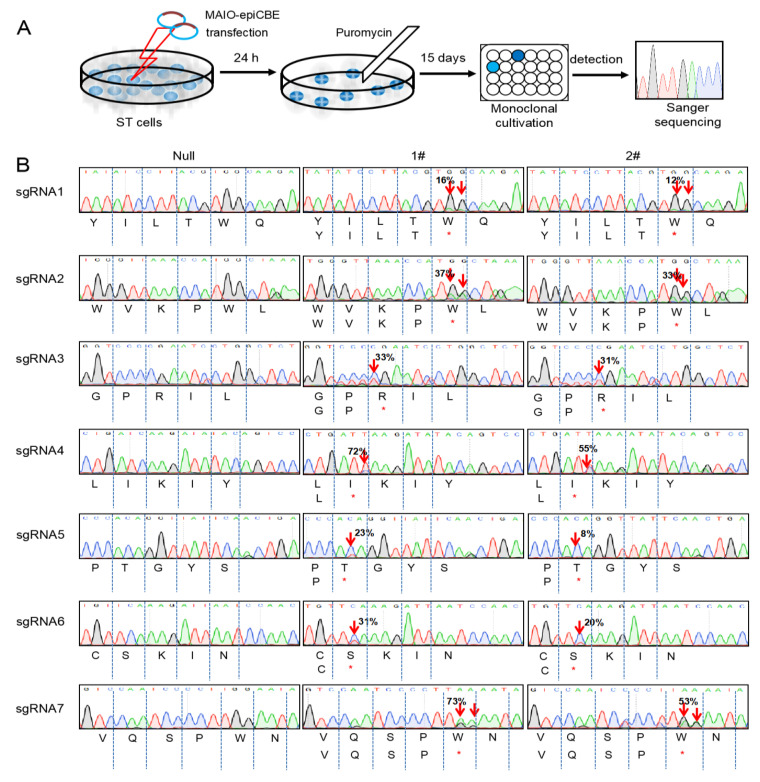
Efficient base editing of PERV genome by MAIO-epiCBE system. (**A**) Schematic of selection for PERV genome-edited clones. (**B**) Base conversion efficiency of clone #1 and #2 detected by Sanger sequencing. All seven target sites were detected and compared to cells transfected with null group. The positions of base substitutions are marked with red arrows. The amino acid sequence and base conversion efficiency are listed under sequence map. * means stop codon site.

Porcine ST cells, the fibroblast-like cells isolated from the testis of a male pig, were used for PERV genome editing. First, ddPCR was performed and about 52 copies of the PERV genome were detected, scattered throughout the ST cells (Supplementary Figure S3). MAIO-epiCBE plasmids were transfected into the ST cells, followed by a single cell culture and puromycin selection for 15 days (Figure 2A). A total of 157 clones were chosen for further culture and analysis. Two clones were chosen for Sanger sequencing. C-to-T or G-to-A base conversion occurred in all seven targets in both cell lines with an efficiency range from 8% to 73% (Figure 2B). All base conversions led to the premature termination of translation.

The Hi-TOM platform was further used for the detection of PERV-editing efficiency in each cell clone. Since the platform determined paired-end reads of 150 bp, three sgRNA target sites on *gag* (sgRNA1, sgRNA2 and sgRNA3) and *pol* (sgRNA4, sgRNA6 and sgRNA7) were analyzed.

**Figure 3:**
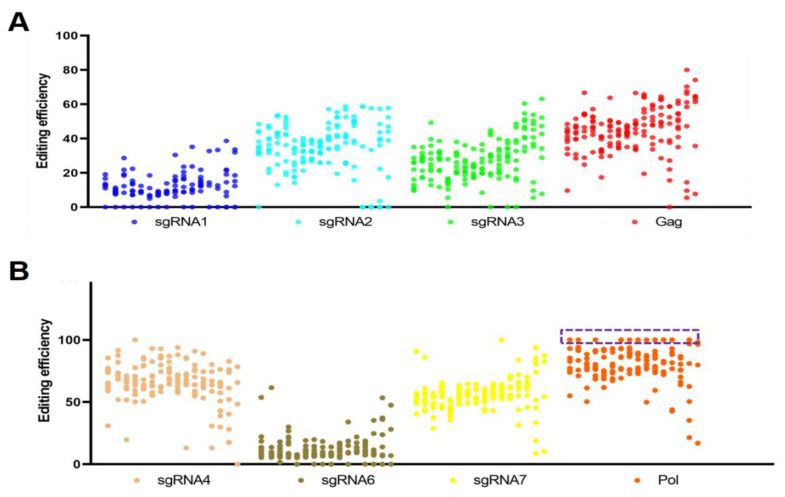
Mi-seq sequencing results of six sgRNA-edited regions on PERV. (**A**) Statistical results of Mi-seq sequencing of sgRNA1, sgRNA2, sgRNA3 and *gag* region from 157 cell clones. (**B**) Statistical results of Mi-seq sequencing of sgRNA4, sgRNA6, sgRNA7 and *pol* region from 154 cell clones. The cell clones with the 100% editing efficiency for all the copies of the *pol* region are boxed in a purple frame.

The statistical results of the 157 clones showed that sgRNA4 was the highest in mutation efficiency while the sgRNA1 was the lowest (Figure 3, Supplementary Table S5). A total of 32.48% cell clones showed more than 50% editing efficiency for all the copies of the *gag* region, while 27.92% cell clones showed more than 90% editing efficiency for all the copies of the *pol* region (Figure 3, Supplementary Table S5). Surprisingly, 11.04% cell clones showed 100% editing efficiency for all the copies of the *pol* region (Figure 3, Supplementary Table S5). These results demonstrated that our MAIO-epiCBE plasmids could be a useful tool to inactivate all copies of the PERV in a single dose of treatment.

### 3.3. The Safety of MAIO-epiCBE Vector

**Figure 4:**
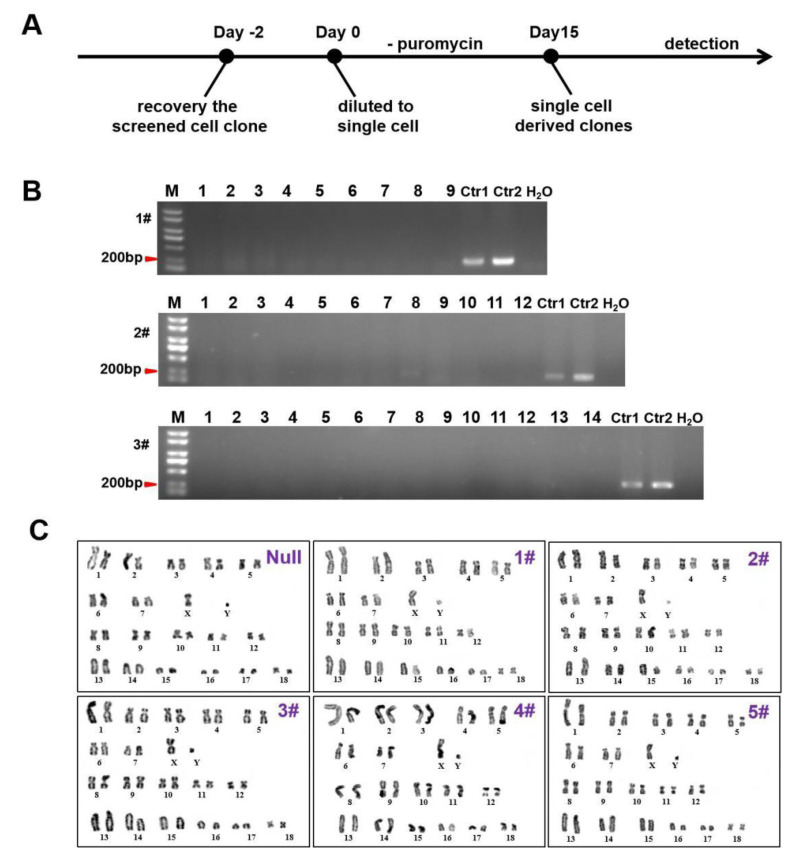
The MAIO-epiCBE plasmid did not integrate into host cells and influence the karyotype of modified cells. (**A**) Schematic of eliminating the MAIO-epiCBE plasmid by withdrawal of puromycin. (**B**) The absence of MAIO-epiCBE plasmid in single-cell-derived clones was demonstrated by PCR with primers: episomal-F and episomal-R. Ctrl1 is the positive control amplified from MAIO-epiCBE vector genome with primers: episomal-F and episomal-R. Ctrl2 is the positive control amplified from the genome of ST cell containing the MAIO-epiCBE plasmid. (**C**) The karyotype analysis of null and five ST cell clones.

To determine whether the MAIO-epiCBE vectors could be removed from the host cells, three ST cell clones were chosen for further analysis. They were disassociated for monoclonal cultivation and cultured for 15 days after puromycin was removed (Figure 4A). None of the subclones were detected to have foreign vectors after the removal of puromycin (Figure 4B). Moreover, five clones were chosen for karyotype analysis. Compared with the null cells, the modified cells did not induce any new detectable karyotype abnormalities (Figure 4C). In summary, the MAIO-epiCBE plasmid did not integrate into the host genome and influence the karyotype of modified cells.

## 4. Discussion

PERVs are integrated into the genomes of all pigs with dozens of genomic copies. Since PERVs can infect human cells [7,8], a potential risk exists in pig-to-human xenotransplantation. Using gene-editing technology to completely knockout PERVs is an effective way to eliminate the expression of pig PERVs. However, ZFN [16] or CRISPR/Cas9 [17,18] technology have usually been found to be toxic to the transfected cells. The induced cytotoxicity is due to the specific cutting of the high copy number of the PERVs in the pig genome.

In the present study, we used the cytosine base editor to edit the targeted sites in which the stop codon was introduced in the region of *gag* or *pol* in pig PERVs. The base-editor system can reduce the double-strand-break-induced cytotoxic effects. Furthermore, it has been reported that PERV inactivation by CRISPR/Cas9 does not compromise virus assembly [29]. Our method tried to cause premature termination codon mutations that would have theoretically resulted in the production of a truncated polypeptide. Furthermore, as the PERVs are integrated with dozens of copies into the genomes of all pigs and the sequences of PERVs have high variation, it is difficult to inactivate PERVs with only a single sgRNA. We thus adopted a multiple sgRNA strategy to facilitate the base-editing-mediated i-stop. Seven sgRNAs that can modify the target sequences to generate stop codon were designed to recognize and edit the region of *gag* or *pol* in the pig PERVs. To improve the efficiency and safety of the editing system, all the functional elements were assembled into non-integrated episomal systems. The cell-editing result indicated that all the seven sgRNAs were able to induce the expected mutations, but the editing efficiencies were different among these sgRNAs. The sgRNA4 showed the highest editing efficiency (74.48%), which may be because of the sequence of the sgRNA4 is located in the highly conserved catalytic center of PERVs. Surprisingly, the multiple sgRNA strategy could induce 100% editing efficiency for all the copies of the *pol* region in 11.04% of cell clones. Many studies have shown that the *env* of PERVs is able to express and form proteins and perform important functions (e.g., syncytium protein). In the follow-up study, we can try to replace the low-editing-efficiency sgRNAs (such as sgRNA1, sgRNA5 and sgRNA6) with the sgRNAs in the *env* of PERVs.

The results also indicated that our editing plasmids did not integrate into the host genome and affect the karyotype of modified cells. However, it is better to examine whether the inactivation of PERVs in the pig genome could eliminate the in vitro transmission of PERVs from pig to human cells. Our results showed that neither WT ST cells nor PERV-edited ST cells transmitted the genome of PERVs into the HEK293 cells (Supplementary Figure S3). It may be because PERVs in ST cells have no reverse transcriptase (RT) activity [29]. Therefore, an infection transmission experiment could be done in the future with other more suitable cell lines. This is the first attempt to use multiple sgRNAs and a cytosine-base-editor-mediated i-stop to terminate PERV gene expression. Taken together, we developed an efficient and safe method for editing pig PERVs. This method may be used to generate PERV-free somatic cells, then further to obtain PERV-free transgenic pigs by somatic cell nuclear transfer technology.

## Data Availability

All data used in this paper are available in the article and Supplementary Materials.

## Supplementary Materials

The following supporting information can be downloaded at: https://www.mdpi.com/article/10.3390/cells11243975/s1, Figure S1: Schematic overview of the procedure for construction the MAIO-epiCBE plasmid; Figure S2: The 4 types of the Hi-TOM platform sequencing results for each ST cell clone; Figure S3: The copy number of PERVs in the five different samples based on the ddPCR result. Table S1: The primers used for amplification the sgRNA fragments; Table S2: The sequence of U6-gRNA-gag, U6-gRNA-pol1 and U6-gRNA-pol2; Table S3: Genomic PCR and sequencing primers; Table S4: Primers for droplet digital PCR; Table S5: Statistical results of Mi-seq sequencing of sgRNA1, sgRNA2, sgRNA3, sgRNA4, sgRNA6, sgRNA7, *gag* and *pol* gene from 157 cell clones.
