# Supplementary material for: Efficient and Safe Editing of Porcine Endogenous Retrovirus Genomes by Multiple-Site Base-Editing Editor
Source: Cells. 2022 Dec 8;11(24):3975. doi: 10.3390/cells11243975 (PMC9776866; doi:10.3390/cells11243975)
Supplement: Supplementary file 1 [file cells-11-03975-s001.zip › cells-1921140-supplementary.pdf]

Supplementary Materials

Efficient and Safe Editing of Porcine Endogenous Retrovirus Genomes by Multiple-Site Base-Editing Editor

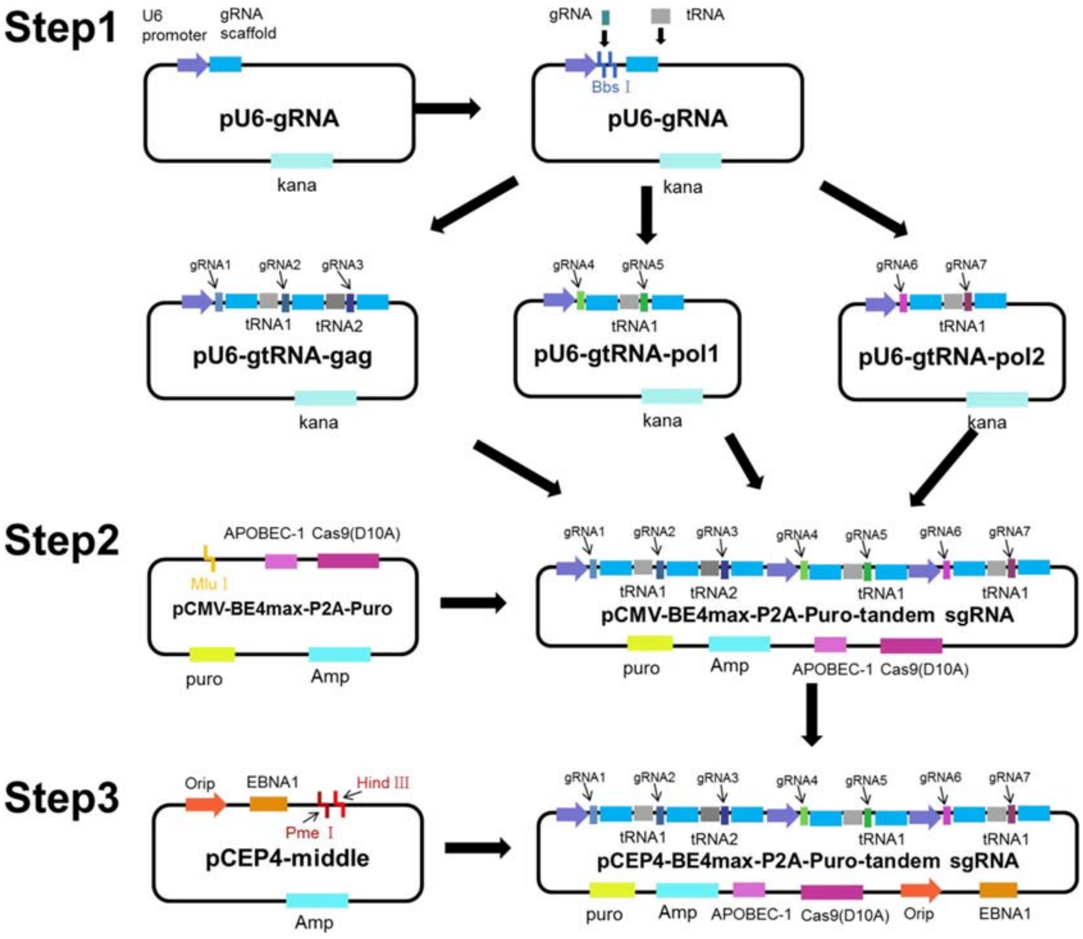

Figure S1. Schematic overview of the procedure for construction the MAIO-epiCBE plasmid.

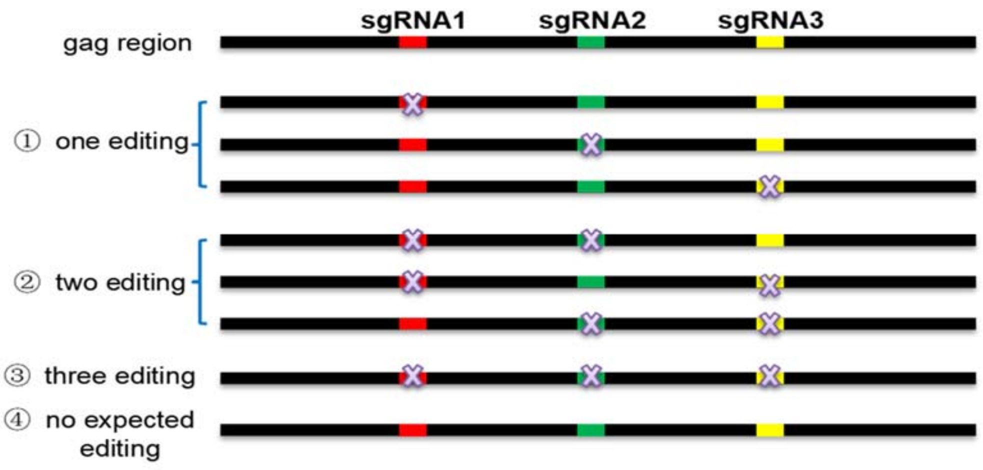

Figure S2. The 4 types of the Hi-TOM platform sequencing results for each ST cell clone.

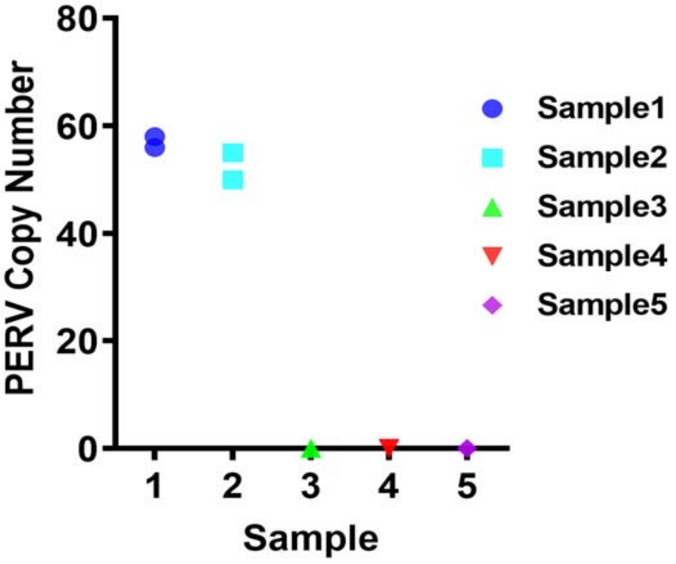

Figure S3. The copy number of PERVs in the five different samples based on the ddPCR result. Each sample has two replicates. Sample1: The digested genomic from PK15 cells; Sample2: The digested genomic from ST WT cells; Sample3: The digested genomic from sorted HEK293-GFP cells of HEK293-GFP/ST WT co-culture cells; Sample4: The digested genomic from sorted HEK293-GFP cells of HEK293-GFP/ST edited clone 1# cells; Sample5: The digested genomic from sorted HEK293-GFP cells of HEK293-GFP/ST edited clone 2# cells. Porcine GAPDH was used as reference gene to calculate the exact copy number of PERV.

Table S1. The primers used for amplification the sgRNA fragments.

Primer name	Primer sequence (5′ to 3′)
PERV-Primer1	ATCTTGTGGAAAGGACGAAACACCGGTCCCCGAATCCTGGCTCTGTTTT AGAGCTAGAAATAGC
PERV-Primer2	CTCGGATCGCTGGATTCAAAGTCCAGAGTGCTAACCATTACAC-CATGGG ACCCTGCTGAAGCACCGACTCGGTGCCAC
PERV-Primer3	CTTTGAATCCAGCGATCCGAGTTCAAATCTCGGTGGGACCTTCTTGCCA CGTAAGGATATAGTTTTAGAGCTAGAAATAGC
PERV-Primer4	GCTATTTCTAGCTCTAAAACTGGGTTAAACCATGGCTAAATGCACCAGC CGGGAATCGAACCCGGGTCTGTACCGTGGC
PERV-Primer5	CCGGGTCTGTACCGTGGCAGGGTACTATTCTACCACTAGACCACTGGTG CTTTGTTAAGCACCGACTCGGTGCCAC
PERV-Primer6	TATCTTGTGGAAAGGACGAAACACCGTATTCCAAGGGGATCGGACGTTT TAGAGCTAGAAATAGCA
PERV-Primer7	GCTATTTCTAGCTCTAAAACGGACTGTATATTTGATCAGAGGTCCCACC GAGATTTGAAC
PERV-Primer8	TATCTTGTGGAAAGGACGAAACACCGTGTTCAAAGATTAATCCAACGTT TTAGAGCTAGAAATAGCA
PERV-Primer9	GCTATTTCTAGCTCTAAAACTCAGCTGAATAACCTGTGGGAGGTCCCAC CGAGATTTGAAC

Table S2. The sequence of U6-gRNA-gag, U6-gRNA-pol1 and U6-gRNA-pol2.

Name	Sequence (5′ to 3′), the red color indicated the sgRNA sequence, the yellow color indicated the tRNA sequence
U6-gRNA-gag	GAGGGCCTATTTCCCATGATTCCTTCATATTTGCATATACGATA-CAAGGCTGTTAGAGAGA-TAATTAGAATTAATTTGACTGTAAACACAAAGATATTAGTACAAAATA

	CGTGACGTAGAAAGTAATAATTTCTTGGGTAGTTTGCAG-<br>TTTTAAAATTATGTTTTAAAATGGACTATCATATGCTTACCGTAACTTGA<br>AAGTATTTCGATTTCTTGGCTTTATATATCTTGTGGAAAGGAC-<br>GAAACAC-<br>CGGTCCCCGAATCCTGGCTCTGTTTTAGAGCTAGAAATAGCAAGTTAA<br>AATAAGGCTAGTCCGTTATCAACTTGAAAAAGTGGCACCGAG-<br>TCGGTGCTTCAG-<br>CAGGGTCCCATGGTGTAATGGTTAGCACTCTGGACTTTGAATCCAGCGA<br>TCCGAGTTCAAATCTCGGTGGGACCTTCTTGCCACGTAAGGATA-<br>TAGTTTTAGAGCTAGAAA-<br>TAGCAAGTTAAAATAAGGCTAGTCCGTTATCAACTTGAAAAAGTGGCA<br>CCGAGTCGGTGCTTAACAAAGCACCAGTGGTCTAGTGGTAGAA-<br>TAGTACCCTGCCACGG-<br>TACAGACCCGGGTTCGATTCCCGGCTGGTGCATTTAGCCATGGTTTAAC<br>CCAGTTTTAGAGCTAGAAATAGCAAGTTAAAATAAGGCTAG-<br>TCCGTTATCAACTTGAAAAAGTGGCACCGAGTCGGTGC
U6-gRNA-pol1	GAGGGCCTATTTCCCATGATTCCTTCATATTTGCATATACGATA-<br>CAAGGCTGTTAGAGAGA-<br>TAATTAGAATTAATTTGACTGTAAACACAAAGATATTAGTACAAAATA<br>CGTGACGTAGAAAGTAATAATTTCTTGGGTAGTTTGCAG-<br>TTTTAAAATTATGTTTTAAAATGGACTATCATATGCTTACCGTAACTTGA<br>AAGTATTTCGATTTCTTGGCTTTATATATCTTGTGGAAAGGAC-<br>GAAACACCG-<br>TATTCCAAGGGGATCGGACGTTTTAGAGCTAGAAATAGCAAGTTAAAA<br>TAAGGCTAGTCCGTTATCAACTTGAAAAAGTGGCACCGAG-<br>TCGGTGCTTCAG-<br>CAGGGTCCCATGGTGTAATGGTTAGCACTCTGGACTTTGAATCCAGCGA<br>TCCGAGTTCAAATCTCGGTGGGACCTCTGATCAAAATATACAG-<br>TCCGTTTTAGAGCTAGAAA-<br>TAGCAAGTTAAAATAAGGCTAGTCCGTTATCAACTTGAAAAAGTGGCA<br>CCGAGTCGGTGC
U6-gRNA-pol2	GAGGGCCTATTTCCCATGATTCCTTCATATTTGCATATACGATA-<br>CAAGGCTGTTAGAGAGA-<br>TAATTAGAATTAATTTGACTGTAAACACAAAGATATTAGTACAAAATA<br>CGTGACGTAGAAAGTAATAATTTCTTGGGTAGTTTGCAG-<br>TTTTAAAATTATGTTTTAAAATGGACTATCATATGCTTACCGTAACTTGA<br>AAGTATTTCGATTTCTTGGCTTTATATATCTTGTGGAAAGGAC-<br>GAAACACCGTGTTCAAA-<br>GATTAATCCAACGTTTTAGAGCTAGAAATAGCAAGTTAAAATAAGGCT<br>AGTCCGTTATCAACTTGAAAAAGTGGCACCGAGTCGGTGCTTCAG-<br>CAGGGTCCCATGGTG-<br>TAATGGTTAGCACTCTGGACTTTGAATCCAGCGATCCGAGTTCAAATCT<br>CGGTGGGACCTCCCACAGGTTATTCAGCTGAGTTTTAGAGCTAGAAA-<br>TAGCAAGTTAAAA-<br>TAAGGCTAGTCCGTTATCAACTTGAAAAAGTGGCACCGAGTCGGTGC

Table S3. Genomic PCR and sequencing primers.

Primer name	Primer sequence (5′ to 3′)
gag-F1	GGCAGACTTTCTGTGCCTCTGA
gag-R1	GCAGCGGTAATATCGCGATCT
pol-F1	GGTAGAGACTTACTGACCAAGA
pol-R1	CGCTCAAGAGGTTATAAGGGT
M-gag-F1	GGAGTGAGTACGGTGTGCTCAGACTGGACCCGGCTCTCAT
M-gag-R1	GAGTTGGATGCTGGATGGCCGAGTGTTTGTTTTTCTCTCCA
M-pol-F1	GGAGTGAGTACGGTGTGCTTCTCCCCTAGTAAAGCCTG
M-pol-R1	GAGTTGGATGCTGGATGGCATTGGTCCCAGGCTTCCTA
episomal-F	AAGCATCGTGGTCAAGGAGGTT

episomal-R	TCTCCGTCATCTCCGTCATCAC

Table S4. Primers for droplet digital PCR.

Primer name	Primer sequence (5′ to 3′)
Pol1-FW	CGACTGCCCCAAGGGTTCAA
Pol-RV	TCTCTCCTGCAAATCTGGGCC
ProbePol	/56FAM/CACGTACTGGAGGAGGGTCACCTG
pig_GAPDH_F	CCGCGATCTAATGTTCTCTTTC
pig_GAPDH_R	TTCACTCCGACCTTCACCAT
Probepig_GAPDH	/5Hex/CAGCCGCGTCCCTGAGACAC

Table S5. Statistical results of miseq sequencing of sgRNA1, sgRNA2, sgRNA3, sgRNA4, sgRNA6, sgRNA7, *gag* and *pol* region from 157 cell clones.

Editing efficiency (%)	sgRNA1 (%)	sgRNA2 (%)	sgRNA3 (%)	sgRNA4 (%)	sgRNA6 (%)	sgRNA7 (%)	Gag (%)	Pol (%)
100								11.04(17/154)
90–100				2.6(4/154)		1.3(2/154)		27.92(43/154)
80–90				12.34(19/154)		5.84(9/154)		25.97(40/154)
70–80				25.32(39/154)		2.6(4/154)	1.91(3/157)	31.17(48/154)
60–70			0.64(1/157)	36.36(56/154)	0.65(1/154)	20.78(32/154)	11.46(18/157)	6.49(10/154)
50–60		13.38(21/157)	3.82(6/157)	12.99(20/154)	1.3(2/154)	43.51(67/154)	19.11(30/157)	2.6(4/154)
40–50		17.83(28/157)	9.55(15/157)	2.6(4/154)	0.65(1/154)	17.53(27/154)	37.58(59/157)	1.95(3/154)
30–40	4.46(7/157)	35.67(56/157)	20.38(32/157)	1.95(3/154)	3.9(6/154)	5.19(8/154)	19.11(30/157)	1.3(2/154)
20–30	5.73(9/157)	15.29(24/157)	36.31(57/157)	1.95(3/154)	5.19(8/154)	0.65(1/154)	5.10(8/157)	1.3(2/154)
10–20	28.66(45/157)	5.10(8/157)	23.57(37/157)	2.6(4/154)	27.27(42/154)	1.3(2/154)	2.55(4/157)	0.65(1/154)
0–10	61.14(96/157)	12.74(20/157)	5.73(9/157)	1.3(2/154)	61.04(94/154)	1.3(2/154)	3.18(5/157)	0.65(1/154)
